# Laboratory and Non-laboratory Assessment of Anaerobic Performance of Elite Male Wheelchair Basketball Athletes

**DOI:** 10.3389/fpsyg.2019.00514

**Published:** 2019-03-13

**Authors:** Jolanta Marszałek, Andrzej Kosmol, Natalia Morgulec-Adamowicz, Anna Mróz, Karol Gryko, Aija Klavina, Kestutis Skucas, José Antonio Navia, Bartosz Molik

**Affiliations:** ^1^Department of Rehabilitation, Józef Piłsudski University of Physical Education in Warsaw, Warsaw, Poland; ^2^Department of Physical Education, Józef Piłsudski University of Physical Education in Warsaw, Warsaw, Poland; ^3^Department of Sport Medicine and Physiotherapy, Latvian Academy of Sports Education, Riga, Latvia; ^4^Department of Applied Biology and Rehabilitation, Lithuanian Sports University, Kaunas, Lithuania; ^5^Facultad de Ciencias de la Actividad Física y del Deporte, Universidad Politécnica de Madrid, Madrid, Spain

**Keywords:** Paralympic sport, anaerobic capacity, field-based testing, wheelchair basketball players, classification in sport, useful assessment tool, adaptive sports

## Abstract

Wheelchair basketball is an adaptive Paralympic sport and wheelchair basketball players are under classification in sport. Coaches are looking for useful assessment tools (field-based tests) to evaluate players’ anaerobic performance (anaerobic capacity). The aim of this study was to assess the validity of field-based tests for anaerobic performance evaluation for two functional categories of wheelchair basketball players and to create a calculator to predict mean or peak power on the basis of the selected field-based test results. Sixty-one elite male wheelchair basketball players performed the Wingate Anaerobic Test and the following field-based tests: 3 m sprint, 5 m sprint, 10 m sprint, 20 m sprint, basketball chest pass test, medicine ball (3 kg) chest pass test, bilateral handgrip, 3-6-9 m drill test, 30-s sprint test, agility drill test and 10 × 5 m sprint test. The participants were divided into two functional categories: A (classes from 1.0 to 2.5; *n* = 29) and B (classes from 3.0 to 4.5; *n* = 32) according to the International Wheelchair Basketball Federation rules. The large effect size (Cohen’s *d* > 0.5) was found in four tests (3 m sprint, 5 m sprint, basketball chest pass test, medicine ball chest pass test; ES 0.90, 0.53, –0.96, –1.05). There were differences between category A and category B players regarding mean power, peak power and relative peak power. Peak power correlated with four tests, while mean power correlated with eight out of eleven tests. The formulas for estimating peak power or mean power in category A and B players were created separately. All the analyses confirmed that 3 m sprint, 5 m sprint, 10 m sprint, 20 m sprint, agility drill test, bilateral handgrip, 3-6-9 m drill test, 30-s sprint test, basketball chest pass test and medicine ball chest pass test are valid for non-laboratory anaerobic performance evaluation. Using the four formulas as a tool to predict mean or peak power on the basis of the selected field-based test results and functional categories will be helpful and will allow coaches and players to prepare pre-season, post-season and in-season conditioning exercises in wheelchair basketball.

## Introduction

Wheelchair basketball is a high-profile Paralympic sport. Rules of wheelchair basketball are similar to those in “running” basketball and are described by the International Wheelchair Basketball Federation (IWBF), 2018). The players with different physical impairments are divided into functional classes (International Wheelchair Basketball Federation, 2014). There are five major functional classes: 1.0, 2.0, 3.0, 4.0 and 4.5 (a higher class denotes a higher level of functional abilities on the court). Furthermore, players with functional capabilities of two neighboring classes can be classified as 1.5, 2.5 or 3.5. The sum of points of five players in one team on the court cannot exceed 14 (International Wheelchair Basketball Federation (IWBF), 2018). Players can be classified to category A (1.0 to 2.5) or category B (3.0 to 4.5) (International Wheelchair Basketball Federation, 2014).

The assessment of physical fitness in wheelchair basketball players is important in order to evaluate their physical state. Previous studies have shown that wheelchair basketball players perform intermittent efforts in the game and indicated an important role of short-time maximal-intensity efforts (Coutts, 1992; Goosey-Tolfrey, 2005; Hutzler et al., 2000). Coutts (1992) suggested that wheelchair basketball players required aerobic as well as anaerobic performance (e.g., during an attack, in defense and playing with the ball). Hutzler et al. (2000) concluded that anaerobic performance depended on the efficiency of wheelchair basketball players on the court. Goosey-Tolfrey (2005) indicated that short-term efforts are very important for wheelchair basketball players and an improvement in anaerobic performance could affect players’ abilities on the court. This author also underlined the fact that an improvement in anaerobic performance is significant particularly for low-category players due to their trunk instability. There are several studies which introduced and explained the specificity of wheelchair basketball intensity (Croft et al., 2010; Iturricastillo et al., 2016; Iturricastillo et al., 2018; Mason et al., 2018; Marszalek et al., 2019). For instance, Croft et al. (2010) underlined that the specificity of wheelchair basketball required high-intensity efforts, e.g., in shooting, dynamic maneuvering or rebounds, and training these skills with high intensity would be more beneficial for players. Mason et al. (2018) highlighted the fact that wheelchair basketball players should practice more 3 vs. 3 small-sided games on half a court to practice high-intensity technical skills like turnovers, rotations, rebounds and shots more efficiently. Iturricastillo et al. (2016) observed high maximum heart rate (HRpeak) in wheelchair basketball games, which means that wheelchair basketball is a demanding sport. They also noted that the rate of perceived exertion (RPE) is the most useful method of assessing match load. Iturricastillo et al. (2018) showed that playoff wheelchair basketball matches were more demanding than league matches. Marszalek et al. (2019) investigated the percentage time contribution of elite players in five heart rate zones during a basketball game. It turned out that players spent 65% of game time in three heart rate zones (60–69%, 70–79% and 80–89% HRpeak). Compared to players from category B, players from category A spent less time in the fifth heart rate zone (90–100% HRpeak; 15 vs. 21%). This study also confirmed intermittent efforts in wheelchair basketball.

Research has revealed certain relationships between classification levels and athletes’ anaerobic performance (Hutzler et al., 1998; Molik et al., 2006, 2010a,b, 2013; de Lira et al., 2010). For instance, de Lira et al. (2010) observed correlations between functional classification of players and their level of anaerobic performance in terms of peak power (PP), relative peak power (rPP) and mean power (MP). The authors confirmed that the functional classification in wheelchair basketball depends on players’ ability on the court and their levels of anaerobic performance. Taking into account PP in the Wingate Anaerobic Test (WAnT), Hutzler et al. (1998) divided male wheelchair basketball players into three groups: high-level paraplegia, low-level paraplegia (category A) and amputation of lower limbs (category B). Molik et al. (2010a,b) compared Polish and Lithuanian wheelchair basketball players’ anaerobic performance (results in the WAnT and in six field-based tests) across all eight classification levels. The level of anaerobic performance demonstrated by athletes in classification category A (functional classes 1.0 to 2.5) was significantly lower compared to category B (3.0 to 4.5), whereas differences between neighboring classes were not found. Also, differences between categories A and B in the results of the WAnT have been found in other studies carried out on male Polish league players and elite female players (Canadian Wheelchair Basketball Team) (Molik et al., 2006, 2013). However, Yanci et al. (2015) did not report significant differences between category A and B in field-based tests such as sprint (5–20 m with and without ball), agility tests (*T*-test and pick-up) and strength tests (handgrip and maximal pass).

In the literature, the Wingate Anaerobic Test (WAnT) is the most popular high-intensity test used for athletes with physical impairments. Accordingly, this test has previously been used among wheelchair basketball players to determine anaerobic performance (PP, rPP, MP, rMP and the fatigue index – FI) (Hutzler et al., 1998; Vanlandewijck et al., 1999; Goosey-Tolfrey, 2005; Hutzler et al., 2000; Molik et al., 2006, 2010b, 2013; de Lira et al., 2010).

It would be useful for practitioners to assess players’ anaerobic performance using easy and feasible field-based tests, not only laboratory tests. For instance, Vanlandewijck et al. (1999) showed correlations between anaerobic performance and field tests – layup, figure-eight + ball, 20 m sprint, zone-shot, figure-eight, pass for accuracy. The authors concluded that the field-based battery of tests is reliable and valid for male wheelchair basketball players with respect to the parameters of anaerobic performance and basketball skill proficiency. Moreover, the authors underlined a strong correlation between the distance covered in the anaerobic field test (30 s sprint) and the WAnT (*r* = 0.93). Molik et al. (2013) selected seven field-based tests: 5 m and 20 m sprint, basketball chest pass test, slalom with the ball, slalom without the ball, shooting accuracy test and bilateral handgrip. The strongest correlation between the WAnT and the field-based test was found for the two-handed chest pass test. This result indicated that the chest pass test can be used to assess anaerobic performance indirectly. De Groot et al. (2012) also confirmed the reliability and validity of selected field-based tests for wheelchair basketball players such as 20 m sprint with ball, picking up the ball, suite, lay-up, spot shot and pass for accuracy. Yanci et al. (2015) confirmed high reliability of the agility *T*-test for the measurements of physical fitness of wheelchair basketball players. Several other researchers used the 20 m sprint test (Traballesi et al., 2009; Chapman et al., 2010; Yanci et al., 2015) or repetitive 15 × 20 m sprints (total time noted) (Goosey-Tolfrey et al., 2010) to measure anaerobic performance of wheelchair basketball players.

In general, findings show inconclusive results regarding differences between two functional categories of wheelchair basketball players. Moreover, previous studies have not looked into the relationships between field and laboratory tests separately for each functional category (A and B). Finally, regression models that would eventually help to predict mean power (MP) or peak power (PP) on the basis of field-based test results have not been developed in previous studies. Therefore, the aim of this study was to assess the validity of field-based tests for anaerobic performance evaluation for two functional categories of wheelchair basketball players and to create a calculator to predict MP or PP on the basis of the selected field-based test results.

## Materials and Methods

### Participants

Sixty-one elite male wheelchair basketball players (mean age 28.5 ± 6.7 years) representing national wheelchair basketball teams of Poland (*n* = 23), Latvia (*n* = 8), Lithuania (*n* = 11) and France (*n* = 19) volunteered to participate in this study. They were informed about the purpose and all testing procedures and were asked to sign the consent form. This study was carried out in accordance with the recommendations of ‘Ethics and Bioethics Committee of the Cardinal Stefan Wyszynski University’ (Komisja Etyki i Bioetyki Uniwersytetu Kardynała Stefana Wyszyńskiego; KEIB – 10/2016) and ‘the Senate Ethics Commission of Jozef Pilsudski University of Physical Education in Warsaw’ (Senacka komisji Etyki Akademii Wychowania Fizycznego Józefa Piłsudskiego w Warszawie; SKE 01-16/2017), with written informed consent from all subjects. All the procedures were approved by the local Bioethics Committees (KEIB – 10/2016, SKE 01-16/2017) and were completed in accordance with the ethical standards as described in the Declaration of Helsinki. Data collection was carried out between February 2017 and July 2018, during training camps of the national wheelchair basketball teams.

The participants were divided into two functional categories: A (classes from 1.0 to 2.5; *n* = 29) and B (classes from 3.0 to 4.5; *n* = 32) according to the IWBF rules (International Wheelchair Basketball Federation, 2014). All the players were evaluated by international classifiers. The health conditions of participating athletes were as follows: spinal cord injury (*n* = 28), spina bifida (*n* = 8), lower limb amputations (*n* = 13), poliomyelitis (*n* = 2), cerebral palsy (*n* = 1) and other physical impairments (*n* = 9).

All individuals were asked about their age and wheelchair basketball training experience. Body mass, upper limb reach in a seated position (in sports wheelchair) and range of upper limbs were measured. The characteristics of wheelchair basketball players are presented in Table 1.

**Table 1:** Characteristics of wheelchair basketball athletes.

Category	Age [years]	Sports experience [years]	Body mass [kg]	Upper limb reach in a seated position [cm]	Range of upper limbs [cm]
Category A (class 1.0–2.5)	27.3 ± 6.6	6.9 ± 4.5	77.4 ± 27.5	179.0 ± 16.6	178.4 ± 36.7
Category B (class 3.0–4.5)	29.3 ± 6.9	8.7 ± 6.3	77.5 ± 14.2	197.7 ± 22.7	188.5 ± 9.3
Total	28.5 ± 6.7	7.2 ± 5.6	77.4 ± 21.2	185.7 ± 22.1	187.7 ± 22.4


### Procedure

#### The Laboratory Test – The Wingate Anaerobic Test

The Wingate Anaerobic Test (the WAnT) was conducted on LODE ANGIO (Groningen, Netherlands) arm crank ergometer (ACE) using the Wingate Anaerobic Software Package – Wingate v.1.07b (Groningen, Netherlands). To maximize the players’ trunk stability, the athletes used their own basketball wheelchairs and strapping. The ACE was firmly fixed to a wall-mounted gymnastic ladder. The axis of rotation of the ergometer was set at the level of the athlete’s glenohumeral joints. To help minimize rotational movements while arm-cranking, the wheelchair itself was stabilized by two assistants.

Each athlete performed one WAnT protocol. The test protocol included a 2-min cranking warm-up at 60 rpm with 50 W resistance for 2 min. Then, resistance was automatically set at the predetermined testing level and the athlete was instructed to crank as fast as possible for 30 s. The software began the 30-s count down as soon as the level of 25 rpm was achieved. Verbal encouragement was given throughout the test. During the assessment of anaerobic performance, the resistance of the ergometer was set on the basis of an individual profile, i.e., 4% of body mass for participants of category A and 5.5% for players belonging to category B.

Four parameters were measured during the WAnT, i.e., peak power (PP) defined as the highest 5-s power output, mean power (MP) defined as the average power sustained throughout the 30-s period, relative peak power (rPP; scaled to individual body mass in kilograms) and relative mean power (rMP; scaled to individual body mass in kilograms).

#### The Non-laboratory Tests – Field-Based Tests

To assess short-term maximal-intensity efforts, the following eleven field-based tests were used: 3 m sprint, 5 m sprint, 10 m sprint, 20 m sprint, basketball chest pass test, medicine ball (3 kg) chest pass test, bilateral handgrip, 3-6-9 m drill test, 30-s sprint test, agility drill test and 10 × 5 m sprint test. Time in all sprint tests was measured with the use of Microgate^®^ photocells (electronic time measurement system with an accuracy of 0.01 s; Bolzano, Italy) and Witty Manager software (version 1.4.1). The participant was seated with the rear wheel axle lined up with the starting line and the timer was activated automatically when the participant was ready to start. All the tests were performed within 1 day with long intervals. Before the testing, all players were asked to do a warm-up for 10 min by themselves (propelling the wheelchair around the court, dynamic stretching of upper limbs and trunk).

**FIGURE 1:**
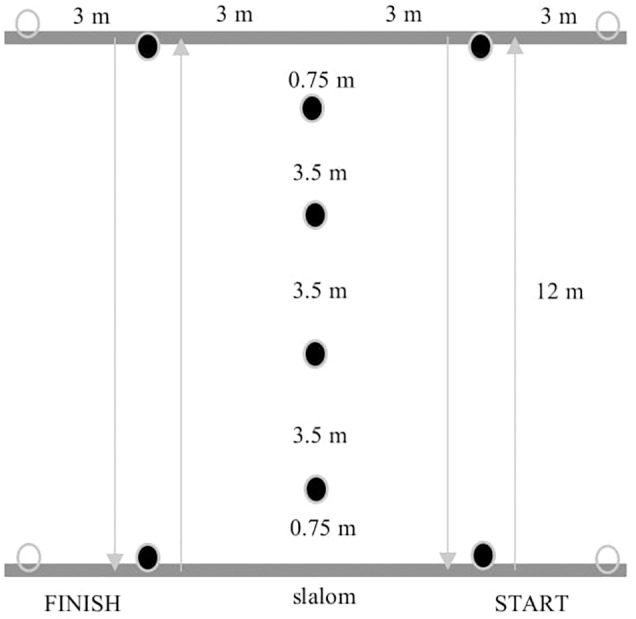
The scheme of the agility drill test (authors’ own interpretation).

**FIGURE 2:**
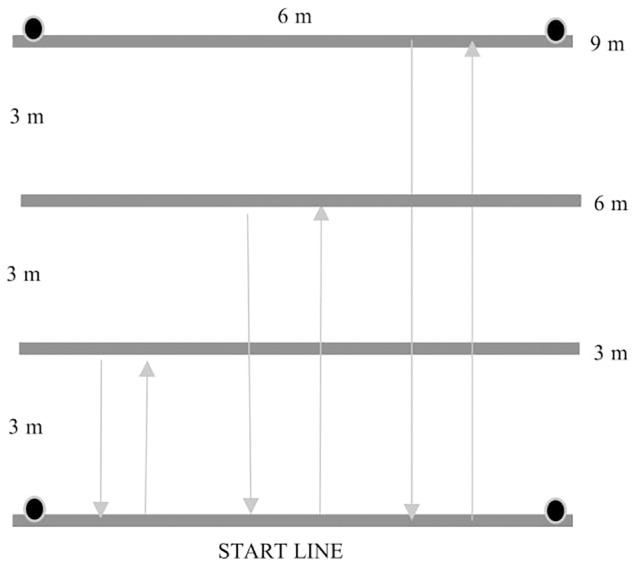
The scheme of the 3-6-9 m drill test (authors’ own interpretation).

The field-based tests were performed according to the following procedure:

- 3 m sprint, 5 m sprint, 10 m sprint, 20 m sprint tests. The participants pushed as hard and as fast as they could over the 3, 5, 10 or 20 m course. The result was the time in seconds (the faster of the two attempts).
- Bilateral handgrip. The participants squeezed a manual handgrip dynamometer DR3 with tensometer WTP003 using software version 3.1. They performed the test seated in their wheelchairs with the tested arm fully extended and not touching the wheelchair. The result was the combination of the value for the right and left hand.
- Basketball chest pass test and medicine ball (3 kg) chest pass test. The participants were in their wheelchairs with their feet placed on the footrest. The rear wheel axle was lined up with the starting line. The participants were encouraged to perform the task using arms as symmetrically as possible. The result was the distance covered by the ball, the best out of three attempts, measured with a tape from the starting line to the place where the ball fell. The measurement error was ±5 cm.
- 30-s sprint test. The participants propelled their wheelchairs as fast as they could over the distance of 20 m, turned and propelled back for 30 s. The result was the achieved distance in meters. There was only one attempt.
- Agility drill test. The participants propelled as fast as they could over the 12-m course in a straight line, came back to start a slalom (four cons) and returned through the slalom. Then, they went straight over the 12-m course and came back (Figure 1). The result was the time in seconds (the faster of the two attempts).
- 3-6-9 m drill test. The participants propelled as fast as they could over the 3-m course and came back to the starting line, then they covered the distance of 6 m and came back to the starting line. Finally, they propelled for 9 m and returned to the starting line (Figure 2). The result was the time in seconds (the faster of the two attempts).
- 10 × 5 m sprint test. The participants propelled as fast as they could 10 times over the 5-m course. The result was the time in seconds (the faster of the two attempts).

### Statistical Analysis

We first compared all the data collected (dependent variables) from categories A and B (factor) using the *t*-test for independent samples. When assumptions of normal distribution (Shapiro–Wilk test) or equal variances (Levene’s test) were violated, the Mann–Whitney *U* test was performed instead. The effect size was displayed with Cohen’s *d*, with 0.2, 0.5, and 0.8 values of d for small, medium and large effects, respectively (Cohen, 1988).

Afterward, we performed a correlation analysis between field and laboratory dependent variables using Pearson’s *r* correlation test for each category (A and B). The level of significance was set at *p* < 0.05 and weak correlation at *r* = 0.1–0.3, moderate correlation at *r* = 0.31–0.5 and strong correlation at *r* > 0.5 (Cohen, 1988). Finally, we developed regression models to estimate the parameters of the WAnT (MP, PP, rMP and rPP) out of all field-test measures for each category separately. Collinearity and autocorrelation were controlled with Tolerance test and Durbin–Watson test, respectively, whereas further assumptions were controlled by means of Q-Q and residual scatterplots. Jamovi software (V.0.9.5.12 for Mac) was used to perform statistical analysis.

## Results

Table 2 presents information regarding the performance of the players and differences between categories in field tests. The large effect size (Cohen’s *d* > 0.5) was found in four tests (3 m sprint, 5 m sprint, basketball chest pass test, medicine ball chest pass test) that showed differences between players from category A and B (Table 2).

**Table 2:** Results and differences in the results of field-based tests performed by wheelchair basketball players from the functional category A and B.

Field-based tests	Category	Mean	Median	*SD*	*SE*	Test	*p*	*d*
3 m sprint [s]	A	1.37	1.36	0.12	0.02	*U*	0.004*	0.90ˆ
	B	1.27	1.27	0.09	0.02			
5 m sprint [s]	A	2.14	1.96	0.73	0.16	*U*	0.004*	0.53ˆ
	B	1.87	1.86	0.15	0.03			
10 m sprint [s]	A	3.28	3.21	0.27	0.05	*U*	0.250	0.19
	B	3.22	3.15	0.32	0.06			
20 m sprint [s]	A	5.57	5.48	0.47	0.10	*U*	0.06	0.37
	B	5.40	5.30	0.48	0.09			
Agility drill test [s]	A	29.26	29.06	2.59	0.57	*U*	0.178	0.20
	B	28.67	28.02	3.26	0.67			
Bilateral handgrip [N]	A	100.70	104.00	29.88	6.23	*U*	0.317	-0.33
	B	109.17	110.00	21.15	4.32			
30-s sprint test [m]	A	99.12	100.00	7.98	1.60	*U*	0.272	-0.30
	B	101.75	101.00	9.35	1.83			
10 × 5 m sprint test [s]	A	22.42	22.47	0.75	0.37	*T*	0.664	0.28
	B	22.13	21.77	1.15	0.43			
3-6-9 m drill test [s]	A	15.22	14.83	1.28	0.28	*U*	0.869	-0.06
	B	15.31	14.92	1.91	0.38			
Basketball chest pass test [m]	A	10.21	10.10	1.47	0.29	*U*	0.001*	-0.96ˆ
	B	12.32	12.20	2.71	0.52			
Medicine ball chest pass test [m]	A	5.90	6.10	0.93	0.19	*T*	0.001*	-1.05ˆ
	B	7.08	7.30	1.27	0.24			


*Category A – class 1.0–2.5; category B – class 3.0–4.5; ^∗^statistically significant difference (p < 0.05);^ˆ^ large effect size (Cohen’s d > 0.50); SD – standard deviation; SE – standard error; T – T-test; U – Mann–Whitney U test.*

Table 3 includes the participants’ performance in WAnT with regard to each category. Differences between categories were found with a large effect size (Cohen’s *d* > 0.5) for MP and PP and rPP (Table 3).

**Table 3:** Results and differences in the results of the Wingate Anaerobic Test (the WAnT) performed by wheelchair basketball players from functional categories A and B.

WAnT parameters	Category	Mean	Median	*SD*	*SE*	Test	*p*	*d*
Mean power (MP) [W]	A	284.04	295.00	40.70	7.83	*U*	0.001*	-0.99ˆ
	B	344.00	314.00	74.95	14.42			
Peak power (PP) [W]	A	530.15	527.00	130.58	25.13	*T*	0.001*	-1.07ˆ
	B	657.04	682.00	104.85	20.18			
Relative mean power (rMP) [W/kg]	A	4.02	4.00	0.67	0.13	*U*	0.188	-0.49
	B	4.47	4.40	1.11	0.21			
Relative peak power (rPP) [W/kg]	A	7.44	7.60	1.79	0.34	*T*	0.001*	-1.07ˆ
	B	8.50	8.20	1.61	0.31			
Fatigue index (FI) [W/s]	A	14.33	14.90	5.53	1.06	*T*	0.034*	-0.59ˆ
	B	17.38	17.40	4.74	0.91			


*Category A – class 1.0–2.5; category B – class 3.0–4.5; ^∗^statistically significant difference (p < 0.05);^ˆ^ large effect size (Cohen’s d > 0.50); SD – standard deviation; SE – standard error; T – T-test; U – Mann–Whitney U test.*

Table 4 shows the correlation matrix between field and WAnT measures for category A and B. For category A players, PP correlated with the results of three tests, i.e., 3 m sprint, 5 m sprint and medicine ball chest pass test, while MP correlated with the results of eight tests (Table 4). For category B players, PP correlated with the results of two tests, i.e., 20 m sprint and medicine ball chest pass test, while MP correlated with the results of seven tests. The results of all WAnT parameters correlated with the results of 3 m sprint and 5 m sprint tests for category A players and with the results of 20 m sprint for category B players (except rPP).

**Table 4:** Correlations between the results of field-based tests and the Wingate Anaerobic Test (the WAnT) performed by wheelchair basketball players from functional categories A and B.

		WAnT parameters							
		Category A				Category B			
Field tests		MP	PP	rMP	rPP	MP	PP	rMP	rPP
3 m sprint [s]	*r*	-0.69ˆ	-0.50	-0.59ˆ	-0.52ˆ	-0.33	0.07	-0.60ˆ	-0.39
	*p*	< 0.001*	0.021*	0.005*	0.015*	0.117	0.733	0.002*	0.057
5 m sprint [s]	*r*	-0.68ˆ	-0.50	-0.67ˆ	-0.58ˆ	-0.36	-0.13	-0.57ˆ	-0.47
	*p*	0.001*	0.025*	0.001*	0.008*	0.081	0.536	0.004*	0.021*
10 m sprint [s]	*r*	-0.44	-0.16	-0.69ˆ	-0.33	-0.46	-0.14	-0.57ˆ	-0.36
	*p*	0.036*	0.464	<0.001*	0.125	0.018*	0.491	0.002*	0.068
20 m sprint [s]	*r*	-0.40	-0.22	-0.66ˆ	-0.40	-0.49	-0.41	-0.44	-0.35
	*p*	0.056	0.302	<0.001*	0.059	0.011*	0.039*	0.024*	0.081
Agility drill test [s]	*r*	-0.53ˆ	-0.13	-0.71ˆ	-0.35	-0.57ˆ	-0.33	-0.43	-0.21
	*p*	0.015*	0.577	<0.001*	0.125	0.004*	0.122	0.039*	0.337
Bilateral handgrip [N]	*r*	0.11	-0.09	0.18	0.04	0.60ˆ	0.35	0.29	-0.00
	*p*	0.640	0.696	0.416	0.851	0.003*	0.100	0.178	0.984
30-s. sprint test [m]	*r*	0.44	0.16	0.57ˆ	0.24	0.57ˆ	0.29	0.51ˆ	0.28
	*p*	0.032*	0.459	0.004*	0.249	0.003*	0.160	0.009*	0.170
10 × 5 m sprint [s]	*r*	0.36	0.57	-0.09	0.08	-0.25	-0.19	-0.76ˆ	-0.77ˆ
	*p*	0.642	0.434	0.911	0.924	0.587	0.683	0.047*	0.042*
3-6-9 drill test [s]	*r*	-0.49	0.05	-0.16	0.19	-0.54ˆ	-0.30	-0.47	-0.26
	*p*	0.029*	0.823	0.510	0.432	0.006*	0.147	0.019*	0.221
Basketball chest pass test [m]	*r*	0.61ˆ	0.06	0.30	-0.11	0.21	0.37	-0.09	-0.08
	*p*	0.001*	0.771	0.149	0.602	0.302	0.065	0.665	0.715
Medicine ball chest pass test [m]	*r*	0.74ˆ	0.44	0.19	0.11	0.54ˆ	0.57ˆ	0.14	0.05
	*p*	<0.001*	0.030*	0.383	0.621	0.005*	0.002*	0.493	0.794


*Category A – class 1.0–2.5; category B – class 3.0–4.5; ^∗^statistically significant Pearson’s correlations (p < 0.05);^ˆ^ strong Pearson’s correlations. MP – mean power [W]; PP – peak power [W]; rMP – relative mean power [W/kg]; rPP – relative peak power [W/kg].*

Table 5 shows the regression models used to estimate MP in the WAnT. For category A, the best tests to estimate MP in the WAnT are 3 m sprint test and medicine ball chest pass test:

**Table 5:** Regression model predicting the influence of field-based tests on the Wingate Anaerobic Test (the WAnT) mean power for categories A and B.

	WAnT parameter – mean power (MP)					
	Category A			Category B		
	*B*	*SE*	*p*	*B*	*SE*	*p*
Intercept	367.46	62.63	0.001	461.42	132.52	0.002
3 m sprint	-142.06	35.27	0.001			
Medicine ball chest pass test	18.81	4.54	0.001			
10 m sprint				-97.47	33.98	0.009
Bilateral handgrip				1.81	0.52	0.003
*p*	0.001			0.001		
*R^2^*	0.74			0.54		
Durbin–Watson	1.94			0.87		


*p < 0.05; category A – class 1.0–2.5; category B – class 3.0–4.5.*

MP in the WAnT of category A players = 367.46 – 142.06 × 3 m sprint test result + 18.81 × medicine ball chest pass test result.

For category B, the best tests to estimate MP in the WAnT are 10 m sprint test and bilateral handgrip test (Table 5):

MP in the WAnT of category B players = 461.42 – 97.47 × 10 m sprint test result + 1.81 handgrip test result.

Table 6 depicts the regression models used to estimate PP in the WAnT. For category A, the best test to estimate PP in the WAnT is 3 m sprint test:

PP in the WAnT of category A players = 1165.77 – 455.08 × 3 m sprint test result.

**Table 6:** Regression model predicting the influence of field-based tests on the Wingate Anaerobic Test (the WAnT) peak power for categories A and B.

	WAnT parameter – peak power (PP)					
	Category A			Category B		
	*B*	*SE*	*p*	*B*	*SE*	*p*
Intercept	1165.77	249.81	0.001	325.10	98.97	0.003
3 m sprint	-455.08	182.00	0.021			
Medicine ball chest pass test				46.65	13.77	0.002
*p*	0.021			0.002		
*R*^2^	0.26			0.32		
Durbin–Watson	1.31			1.31		


*p < 0.05; category A – class 1.0–2.5; category B – class 3.0–4.5.*

For category B, the best test to estimate PP in the WAnT is medicine ball chest pass test (Table 6):

PP in the WAnT of category B players = 325.10 + 46.65 × medicine ball chest pass test result.

## Discussion

The aim of this study was to assess the validity of field-based tests for anaerobic performance evaluation for two functional categories of wheelchair basketball players and to create a calculator to predict MP or PP on the basis of the selected field-based test results. In the first part of this study, the results of field-based tests of players from two functional categories (category A and category B) were compared. Four out of eleven tests, i.e., 3 m sprint, 5 m sprint, basketball chest pass test and medicine ball chest pass test confirmed statistically significant differences between low and high point category wheelchair basketball players.

In the literature of the subject, there are few studies exploring the differences in field-based anaerobic performance between category A and B wheelchair basketball players (Molik et al., 2013). Those studies have some limitations as they do not systematically indicate in which tests players from category A had significantly different results compared to players from category B (e.g., different field-based tests, different number of participants). In our study, we partially confirmed the results presented by Yanci et al. (2015), who did not find differences in 20 m sprint test and handgrip test between players from category A and B. Yet, unlike previous results, in our study wheelchair basketball players showed different results in 5 m sprint test as a function of their category. Yanci et al. (2015) also compared agility test results (*T*-test and pick-up test) in different categories. In this case, the findings of the present experiment confirmed the lack of differences between two functional categories in agility tests (agility drill test, 10 × 5 m sprint test, 3-6-9 m drill test). In this sense, agility tests require very good wheelchair propulsion and maneuverability abilities. Even though our participants were elite athletes, we suggest that the results in agility tests could be more dependent on experience in wheelchair propulsion rather than on the players’ functional capabilities or types of impairments. Therefore, agility tests should be used by coaches and players to develop wheelchair maneuverability skills on a basketball court.

Previous research showed that performance differences between players of category A and B were apparent in almost all tests (except shooting test) (Molik et al., 2013). Hence, while Molik et al. (2013) revealed differences between elite female players in six out of seven tests, results of the present experiment showed differences only in four out of eleven tests, i.e., in 3 m sprint, 5 m sprint, medicine ball chest pass test and basketball chest pass test. It is worth mentioning that in our experiment, the number of participants assessed was higher than in previous studies by Molik et al. (2013) and Yanci et al. (2015) (61 participants versus 16 and 23 participants, respectively).

de Witte et al. (2018) analyzed 15 different tests (activities) in the group of 46 players on a national and international level. Significant differences in the results of 12 m sprint test were confirmed. However, in the present study we did not find differences between the two categories in 10 m sprint test. Other significant differences underlined by de Witte et al. (2018) were found in 180° turn on the spot (left), 3-3-6 m sprint (sprint with two stops), 90°–90° turn on the spot with a stop (left), 90°–90° turn on the spot with a stop (left) and in combinations. The authors did not find differences in ball dribble and rotations test. These types of tests were not included in our analyses due to a strong influence of a wheeling technique and ball control. Our investigations focused more on relationships with power and anaerobic performance. Still, further research is needed to create the best field-based test battery for wheelchair basketball players.

In the second part of this study, the results of the Wingate Anaerobic Test (the WAnT) were analyzed. The findings of our study confirmed the analysis of Molik et al. (2010b) and showed significant differences between both categories (A and B) in anaerobic performance in the WAnT (except rMP). However, Molik et al. (2010b) did not analyze relative but only absolute parameters of MP and PP in the WAnT. The analysis of rMP and rPP could be discussed due to the specificity of impairments of each player, e.g., a player with lower limb amputation and lower limb muscle atrophy weighs less than a player with lower limb length differences. Therefore, the analysis of MP and PP could be more useful when comparing different athletes in Paralympic sports, especially in wheelchair basketball. Apart from this problem, personal periodic analysis of relative parameters could be useful for a coach and a player in pre-season, post-season and in-season conditioning exercises.

In the third part of this study, correlations between the results from field-based tests and the WAnT parameters were presented. The validity of some field-based tests was confirmed. The strong correlations (*r* < 0.5 for *p* < 0.05) were noted between MP in the WAnT and 3 m sprint test, 5 m sprint, agility drill test, basketball and medicine ball chest pass tests for players from category A and between MP and agility drill test, bilateral handgrip, 30-s sprint test, 3-6-9 drill test, and medicine ball chest pass test for players from category B. Moderate (0.3 < *r* < 0.5 for *p* < 0.05) correlations were documented between PP and 3 m sprint, 5 m sprint and medicine ball chest pass test. Our research confirmed moderate validity of 20 m sprint (category B) which was indicated by Vanlandewijck et al. (1999) and De Groot et al. (2012).

A strong correlation between chest pass tests and MP and PP were underlined in the analysis by Molik et al. (2013) (*r* = 0.80 and *r* = 0.82, respectively). Our research confirmed a strong correlation (with MP in the WAnT) for 3 m sprint and 5 m sprint tests (category A) and medicine ball chest pass test (category A and B). Moreover, 3 m sprint and 5 m sprint tests had stronger correlations with MP than any other sprint tests (*r* = –0.69 and *r =* –0.68) for category A players. Surprisingly, 3 m sprint and 5 m sprint did not correlate significantly with the WAnT for category B players, and 20 m sprint tests did not correlate significantly with the WAnT for category A players. It seems that tests measuring MP and short-term efforts that focus on wheelchair acceleration and explosive power are much more effective in wheelchair basketball game for players from category A, probably because trunk function of players from category A is weaker [according to the classification in wheelchair basketball (International Wheelchair Basketball Federation, 2014)].

It is worth highlighting the separate analysis of WAnT parameters by category performed in the present study so that validations of selected field-based tests can be done more accurately according to an impairment degree. In other studies, researchers only reported whether they found or did not find correlations between selected tests and the WAnT, so it is not possible for the reader to find out which WAnT parameters were correlated. In our study, we found that there are other relationships between MP and PP and selected tests (e.g., 3 m sprint and 5 m sprint correlated with PP more strongly 10 m sprint and 20 m sprint tests). It seems that in wheelchair basketball, all PP results could be more useful for coaches and training development because such actions as wheelchair acceleration, playing one-on-one, long-distance passing or shooting are strictly related with PP. Therefore, our approach to a separate analysis should be continued in the future studies.

In the last part of our study, we developed a calculator to predict MP and PP on the basis of the selected field-based test results. All analyses of regression allowed us to create four independent formulas to predict MP and PP for wheelchair basketball players representing two different functional categories (category A and B). The calculation of MP was based on 3 m sprint test, 10 m sprint test, medicine ball chest pass test and bilateral handgrip test. PP prediction was based on 3 m sprint test for category A, and medicine ball chest pass test for category B. These formulas are easy predictors to assess (estimate) anaerobic performance of wheelchair basketball players in the WAnT. Although all formulas significantly predicted the parameters of WAnT (MP and PP), it has to be highlighted that models for category A players were more precise (more variance explained as depicted by R2 values) than for category B players. Thus, it seems that the accuracy of the estimation of the WAnT values depends on the degree of impairment. This is a good question for further research. In any case, the construction of regression models signifies a step forward in literature, as we did not find these types of predictions or ideas (i.e., WAnT estimators on the basis of field-tests) in previous research.

### Study Limitations

There are no reference values for all the tests to compare players, their physical fitness and anaerobic performance level. In wheelchair basketball classification there are eight functional levels of players (classes). We divided our participants into two categories and we could not compare differences between all classes because of a small number of subjects.

## Conclusion

The present study confirmed the validity of field-based tests for anaerobic performance evaluation in the Wingate Anaerobic Test (WAnT). Within category A, the analysis revealed that field tests like 3 m sprint, 5 m sprint, 10 m sprint, agility drill test, 30-s sprint test, 3-6-9 drill test, basketball chest pass test and medicine ball chest pass test are valid for non-laboratory anaerobic performance evaluation of players from category A. Also, 10 m sprint, 20 m sprint, agility drill test, bilateral handgrip, 30-s sprint test, 3-6-9 drill test and medicine ball chest pass test appeared to be effective for non-laboratory anaerobic performance evaluation of players from category B. Moreover, four formulas to estimate mean power (MP) or peak power (PP) on the basis of the selected field-based test results have been presented. In general, present findings will be helpful and will allow coaches and players to prepare pre-season, post-season and in-season conditioning exercises in wheelchair basketball.

## Data Availability

The datasets for this manuscript are not publicly available because of the entry in the agreement of the local Bioethics Committees (KEIB – 10/2016 and SKE 01-16/2017).

## Author Contributions

JM devised the structure of the paper, drafted the manuscript, collected and analyzed the data, and commented on the final version. AKo collected and analyzed the data, and commented on the final version. NM-A, AM, KG, AKl, and KS collected and analyzed the data. JN supported statistical analyses and reporting, and commented on the final version. BM devised the structure of the paper, drafted the manuscript, oversaw the whole research process, collected and analyzed the data, and commented on the final version.

## Conflict of Interest Statement

The authors declare that the research was conducted in the absence of any commercial or financial relationships that could be construed as a potential conflict of interest.
